# Observations on *Neotricula aperta* (Gastropoda: Pomatiopsidae) population densities in Thailand and central Laos: implications for the spread of Mekong schistosomiasis

**DOI:** 10.1186/1756-3305-5-126

**Published:** 2012-06-21

**Authors:** Stephen W Attwood, E Suchart Upatham

**Affiliations:** 1 State Key Laboratory of Biotherapy, West China Hospital, West China Medical School, Sichuan University, Chengdu, People’s Republic of China; 2 Department of Zoology, The Natural History Museum, London, United Kingdom; 3 Department of Biology, Faculty of Science, Mahidol University, Bangkok, Thailand; 4 Faculty of Allied Health Sciences, Burapha University, Bangsaen, Chonburi, Thailand

## Abstract

**Background:**

The snail *Neotricula aperta* transmits Mekong schistosomiasis in southern Laos and Cambodia, with about 1.5 million people at risk of infection. Plans are under consideration for at least 12 hydroelectric power dams on the lower Mekong river and much controversy surrounds predictions of their environmental impacts. Unfortunately, there are almost no ecological data (such as long term population trend studies) available for *N. aperta* which could be used in impact assessment. Predictions currently assume that the impacts will be the same as those observed in Africa (i.e., a worsening of the schistosomiasis problem); however, marked ecological differences between the snails involved suggest that region specific models are required. The present study was performed as an initial step in providing data, which could be useful in the planning of water resource development in the Mekong. Snail population density records were analyzed for populations close to, and far downstream of, the Nam Theun 2 (NT2) project in Laos in order to detect any changes that might be attributable to impoundment.

**Results:**

The population immediately downstream of NT2 and that sampled 400 km downstream in Thailand both showed a long term trend of slow growth from 1992 to 2005; however, both populations showed a marked decline in density between 2005 and 2011. The decline in Thailand was to a value significantly lower than that predicted by a linear mixed model for the data, whilst the population density close to NT2 fell to undetectable levels in 2011 from densities of over 5000 m^-2^ in 2005. The NT2 dam began operation in 2010.

**Conclusions:**

The impact of the NT2 dam on *N. aperta* population density could be more complex than first thought and may reflect the strict ecological requirements of this snail. There was no indication that responses of *N. aperta* populations to dam construction are similar to those observed with *Bulinus* and *Schistosoma haematobium* in Africa, for example. In view of the present findings, more ecological data (in particular population density monitoring and surveillance for new habitats) are urgently required in order to understand properly the likely impacts of water resource development on Mekong schistosomiasis.

## Background

### Mekong schistosomiasis

**Figure 1:**
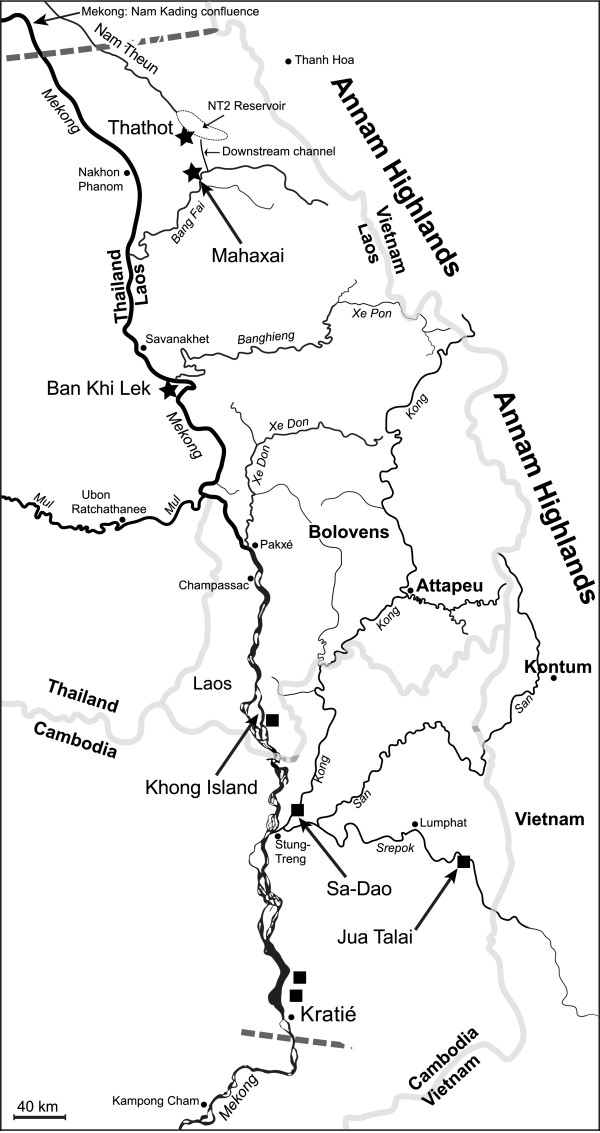
**The lower Mekong Basin, showing the key rivers draining the region.** The northern and southernmost limits of the known range of *Neotricula aperta* are shown by thick dashed lines. Mekong schistosomiasis transmission foci are denoted by black squares and the populations sampled in this study by black stars. The approximate extent of the Nam Theun 2 (NT2) main reservoir is indicated, as is the downstream discharge channel. Scale and international boundaries approximate.

Mekong schistosomiasis occurs in Northeast Cambodia and southern Laos and is a parasitic disease of humans caused by infection with the blood-fluke *Schistosoma mekongi* (Trematoda: Digenea). The freshwater snail *Neotricula aperta* is the sole intermediate host for *S. mekongi *[1]. Although primarily an infection of humans the parasite is also found in pigs and dogs [2,3]. The most studied foci of transmission are those at Khong Island on the Mekong river in Champassac Province of southern Laos (approximately 25 km from the border with Cambodia) [4] and Kratié in Kratié Province, northeastern Cambodia, approximately 180 km downstream of Khong Island [5] (Figure 1). More recently, a transmission focus has been found in the Xe Kong river of northeastern Cambodia at Sa-Dao in Stung-Treng Province, approximately 35 km upstream of the confluence of the Mekong and Xe Kong rivers (Figure 1) [6]. Finally, in 2004, transmission of *S. mekongi* was detected at Jua Talai on the Sre Pok river in Rattanakiri Province, northeastern Cambodia [7]. In Northeast Thailand, three strains of *N. aperta* have been recognized (called α, β and γ), on the basis of shell size and body pigmentation [8]. Although all three strains are susceptible to *S. mekongi* (γ> > β > α), only the γ-strain is epidemiologically significant [9]. At present there are only four known areas of Mekong schistosomiasis transmission and at each of these the γ-strain of *N. aperta* is the only strain found.

Currently, *N. aperta* is known from 31 localities in Cambodia, Lao PDR and Thailand , involving nine river systems [10] and an estimated 1.5 million people are at risk of infection [7]. The known range of *N. aperta* is from just south of Kratié in the Mekong river of Cambodia to Kong Lor in Savannakhet of central Laos. In contrast, the parasite appears to be restricted to the southern tip of Laos, near the border with Cambodia, and northeastern Cambodia, and shows a highly discontinuous distribution (Figure 1). The absence of the parasite from *N. aperta* habitats across southern and central Laos has been attributed to historical factors, in that the parasite may not have had sufficient time to colonize its potential range in Laos following a Pleistocene introduction [10]. There has been some optimism regarding the possible complete control of *S. mekongi* infection [11,12]; however, this may be unfounded, because of the persistence of infection in the snail intermediate host at Khong Island, with relatively unchanged prevalence levels before and after a nine year control programme in Cambodia and Lao PDR. The persistence of infection may be due to survival of the parasite in reservoir hosts [2,3,13]. In spite of the nine years of control efforts, the prevalence of Mekong schistosomiasis in Hat-Xai-Khoun village, Khong Island, is still 26.8 % (but has decreased from almost 80 % in 1989) [1] and at Sa-Dao the prevalence rose from 0 % in 2004 to around 2 % in 2005. Similarly, although no new cases of severe morbidity have been reported in Cambodia since 2002, three new cases of human infection were reported in 2005 [14]; this again highlights the resilience of transmission in the face of seven years of mass chemotherapy (beginning 1996).

### Water resource development in the lower Mekong – a lack of relevant data

Plans for several mainstream dams along the Mekong river in China and Laos are currently under discussion, most notable of these is the 1285 MW Xayaburi Hydro-electric Power Project (HEPP) on the Mekong river mainstream in northern Laos [15], which together with 11 other planned mainstream dams in the lower Mekong have been the subject of much controversy and a challenge for international management [16,17]. In addition to generating electricity, many of these dams are proposed to reduce the perennial problem of severe flooding in riparian areas; for example, the proposed Gonguoqiao Dam on the Mekong river in China is primarily intended for flood control [15]. In the lower Mekong 75 % of the annual flow occurs during the July-October rainy season, with lowest flow rates occurring in April.

The possible worsening of the impact of Mekong schistosomiasis on public health following HEP development in the lower Mekong has been discussed in the recent literature; however, the examples pertaining to schistosomiasis given in published reports on Mekong HEP development are often based on experiences in Africa (involving *Schistosoma mansoni* and *S. haematobium*) [18] or on *Schistosoma japonicum* and schistosomiasis in China [15,19]. In other reports it has been assumed that water resource development in Laos has altered ecological conditions such that an expansion of the range of *S. mekongi* into new areas could occur [20]. In support of these assumptions there are currently very few published data which are relevant to an assessment of the impact of HEPPs on schistosomiasis in this region [21] and relatively little insight can be gained from studies of schistosomiasis in Africa because of the very different transmission ecologies [22]. Clearly there is an urgent need for ecological data which can be used to help assess the likely impact of HEPPs on Mekong schistosomiasis.

### The transmission ecology of *Schistosoma mekongi* – the need for species specific data in impact assessment

Examples of a worsening of schistosomiasis following dam construction in Africa are well known; for example, the Akosombo Dam in Ghana (*S. haematobium*) [23], the Kossou Dam in Côte d'Ivoire (*S. haematobium*) [24], the Oyan River Dam in Nigeria (*S. haematobium*) [25], and the Diama and Manantali Dams in Senegal (*S. mansoni*) [26], all of which have been implicated in effecting at least three-fold increases in schistosomiasis prevalence post-impoundment. The intermediate hosts of the *S. haematobium* and *S. mansoni* groups are all pulmonate snails of the family Planorbidae, species of *Bulinus* (mostly) and *Biomphalaria*, respectively [27]. Planorbid snails have very different habitat requirements from the triculine snails responsible for the transmission of *S. mekongi,* and this leads to very different transmission ecologies in the Mekong relative to the African *Schistosoma* infecting humans. Consequently, the planning of dam projects and prediction of their public health impact in the lower Mekong must be based on ecological studies specific to Mekong schistosomiasis, and the use of any data based on the transmission of other species is unlikely to be appropriate.

Snails of the *Bulinus africanus* group show a wider range of habitat tolerance than *N. aperta*; for example the majority of *B. africanus* habitats (in which persistent populations are found) are lentic habitats or those with a flow rate < 0.3 ms^-1^ and these snails are often found in muddy substrata [28]. Species of *Bulinus* are commonly found in the reservoirs of dams and in fish ponds; these taxa are also relatively tolerant of turbidity and contamination by organic matter (indeed, some species, such as *Bulinus globosus*, have been reported to show an increased population density in habitats polluted by animal feces or rotting vegetation) [29]. Similarly, *Biomphalaria spp.* are often found in lakes, reservoirs of dams, ditches, irrigation channels, fish ponds and rice fields [30,31]– all habitats in which *N. aperta* has not been found. In addition while the planorbid snails discussed here are associated with aquatic macrophytes, *N. aperta* is exclusively epilithic or epixylic (on rotting wood). *Neotricula aperta* is found only in shallow areas (typically 0.5 to 3 m deep) of the Mekong river and some of its tributaries. The snails are restricted to areas where the current is moderate (around 2 x 10^3^ m^3^s^-1^), the water is clear and the bed rock forms (almost flat) platforms where agal aufwuchs is extensive [1]. Such conditions exist only during the dry season in the lower Mekong (March to May) and so *N. aperta* populations persist mostly by recruitment (from eggs laid on stones in the previous year) or re-colonization from other rivers, and transmission of *S. mekongi* is seasonal [32]. *Neotricula aperta* grazes the algal epilithon and therefore, unlike species such as *Biomphlaria*, cannot survive in areas where sediment is depositing and preventing the growth of the algae upon which it feeds. Indeed, ecological studies of *N. aperta* have shown that this snail is found only on stones covered with fine sediments and that this species is highly sensitive to silting [33]. In addition, *N. aperta* is not known from waters of low conductivity or *pH* and is calciphilic; the *pH* of all *N. aperta* habitats sampled to date is > 7.5 and the river systems in which the snails are found have always been those draining karst areas. More recently, *N. aperta* has been found in the primary streams emerging from karst springs, close to the origins of the streams [10]. Similarly, parallels drawn with the situation of *S. japonicum* and *Oncomelania hupensis* in China are of limited relevance. Although *Oncomelania* is a pomatiopsid snail, it is amphibious and occupies different habitats from those of *N. aperta*, such as ditches, drainage channels, rice paddy, wetlands, the shores of lakes and margins of artificial ponds. *Oncomelania* is also much more tolerant of silting and turbid waters [34] and has greater dispersal capabilities [35,36].

In view of the marked ecological differences between *N. aperta* and the pulmonate snails involved in the transmission of *Schistosoma* to humans in Africa and the New World, only ecological data and historical evidence from studies on *N. aperta* should be considered in assessment of the impact of HEPPs on schistosomiasis in the lower Mekong. The ecological requirements of *N. aperta* indicate that this snail will not become established in the reservoirs or downstream channels of dams in the region and that flooding of habitats by impoundment will eliminate all *N. aperta* populations from the affected area. Consequently, predictive studies of the effects of HEPPs should concentrate on the responses of snail populations far downstream. The dams are likely to destroy existing habitats upstream of the impoundment, but reductions in flow rate and attenuation of the natural flood cycle downstream might create new areas suitable for *N. aperta*. In view of this, relevant ecological studies are now urgently required to enable the planning and sighting of HEPPs so as to minimize any negative impacts on the schistosomiasis problem in the lower Mekong.

### Aims of the study

In view of the number of HEPPs now being planned for the lower Mekong, the controversy surrounding them and the lack of data that can be used to guide the planning of these projects, the present study was undertaken as a first step in a scientific assessment of the impact of existing HEPPs on *N. aperta*. The objective of this study was to analyse snail population density estimates available from 1991 until 2011 and to detect any changes in population growth trend that might be attributable to the effects of HEPPs. In order to improve detection of the potential effects of impoundment, snail populations were sampled at sites close to the Nam Theun 2 (NT2) HEPP in Khammouanne Province, central Laos, (17°59′49″N; 104°57′10″E) and from a site over 400 km downstream on the Mekong river in Thailand. The 39 m gravity dam of the NT2 project was closed in March 2010 and the project includes a 450 km^2^ upstream reservoir, an 8 km^2^ artificial regulating pond and a 27 km artificial downstream discharge channel that feeds directly into the Xe Bang Fai river 10.5 km upstream of Mahaxai village (Figure 1). The Thai sampling site lies just downstream of the confluence between the Xe Bang Fai river and the Mekong river, and the sampling sites in Laos are at Mahaxai and at a site near to the regulating pond (Thathot). The three sites are the only locations for which we have long-term population data.

Trends are estimated for the observed time series using a linear mixed model expression of the Gompertz stochastic state-space exponential growth model, with modification to allow for unequally spaced sampling intervals [37,38]. The model incorporates both observation error and environmental process noise and has shown success with shorter time series and uneven sampling times similar to those in the present investigation [37]. Through this sampling and analysis the study aimed to provide the first insight into population growth trends of *N. aperta* in areas expected to be impacted by HEPP operations.

## Results

### Model fitting and parameter estimation

Initial examination of the data revealed that the population density at MHX is clearly much greater than at BKL (over 10 times), with no overlap between the data sets over the time series (Table 1). The observed 2011 population density was an outlying value in both the BKL and MHX field surveys; the observed density in April 2011 at MHX fell to undetectable levels (from 5190 m^-2^ in 2005) and that at BKL fell from 318 m^-2^ in 2005 to 140 m^-2^ in 2011. The effect of inclusion of the outliers can be seen in the much lower standard deviation (among temporal samples) after their removal from the data sets (Table 1). Simple linear regression did not detect any significant correlation between population density and time (i.e., no significant trend) at BKL, either with or without the 2011 observation; however, a significant trend was found at MHX with the outlier excluded (Table 2). Attempts to fit a Poisson GLM failed in all cases except for the BKL data after removal of the outlier (Table 2). A positive trend (i.e., population growth) was found in all cases with these basic models, when the 2011 observations were excluded. At Thathot the population density was also seen to fall in 2011, although only two samples were available (864 m^-2^ 28 March 2004 and 169 m^-2^ 27 April 2011).

**Table 1:** Summary statistics for the time series observations of *Neotricula aperta* population density

**Population**	**Sampling period**	**No. of observations in time series**	**Mean population density m**^**-2**^** ± SD**
BKL (full)	1991 - 2011	8	289.75 ± 59.88
BKL (w/o outlier)	1991 - 2005	7	310.86 ± 5.05
MHX (full)	1992 - 2011	7	4057.00 ± 1840.08
MHX (w/o outlier)	1992 - 2005	6	4734.00 ± 470.34
Thathot	2004 - 2011	2	516.50 ± 491.44

Data are summarized for the whole series of observations and for a subset of the observations with outliers excluded. BKL, Ban Khi Lek, Khemmarat, Northeast Thailand; MHX, Mahaxai, Khammouanne Province, Laos.

**Table 2:** Fit of conventional linear models to the population density estimates for *Neotricula aperta*

**Population**	**Simple Linear Regression**			**General Linear Model (Poisson)**			
	**Equation (Density =)**	**Student's-*****t***	***P***	**Null deviance**	**Residual deviance**	**AIC**	***P***
BKL (full)	−0.1055 t + 342.7367	−1.8540	0.113	104*	72.59	136.4	<0.0001
BKL (w/o outlier)	0.0096 t + 306.7542	1.4520	0.206	0.4918	0.3456	57.39	0.997
MHX (w/o outlier)	1.8038 t + 4056.1090	5.017 *	0.007	241.9 *	38.49	104.30	<0.0001

For the Simple Linear Regression, t represents time in weeks from the first sample (t = 0). A * indicates a statistically significant value for the test statistic.

**Figure 2:**
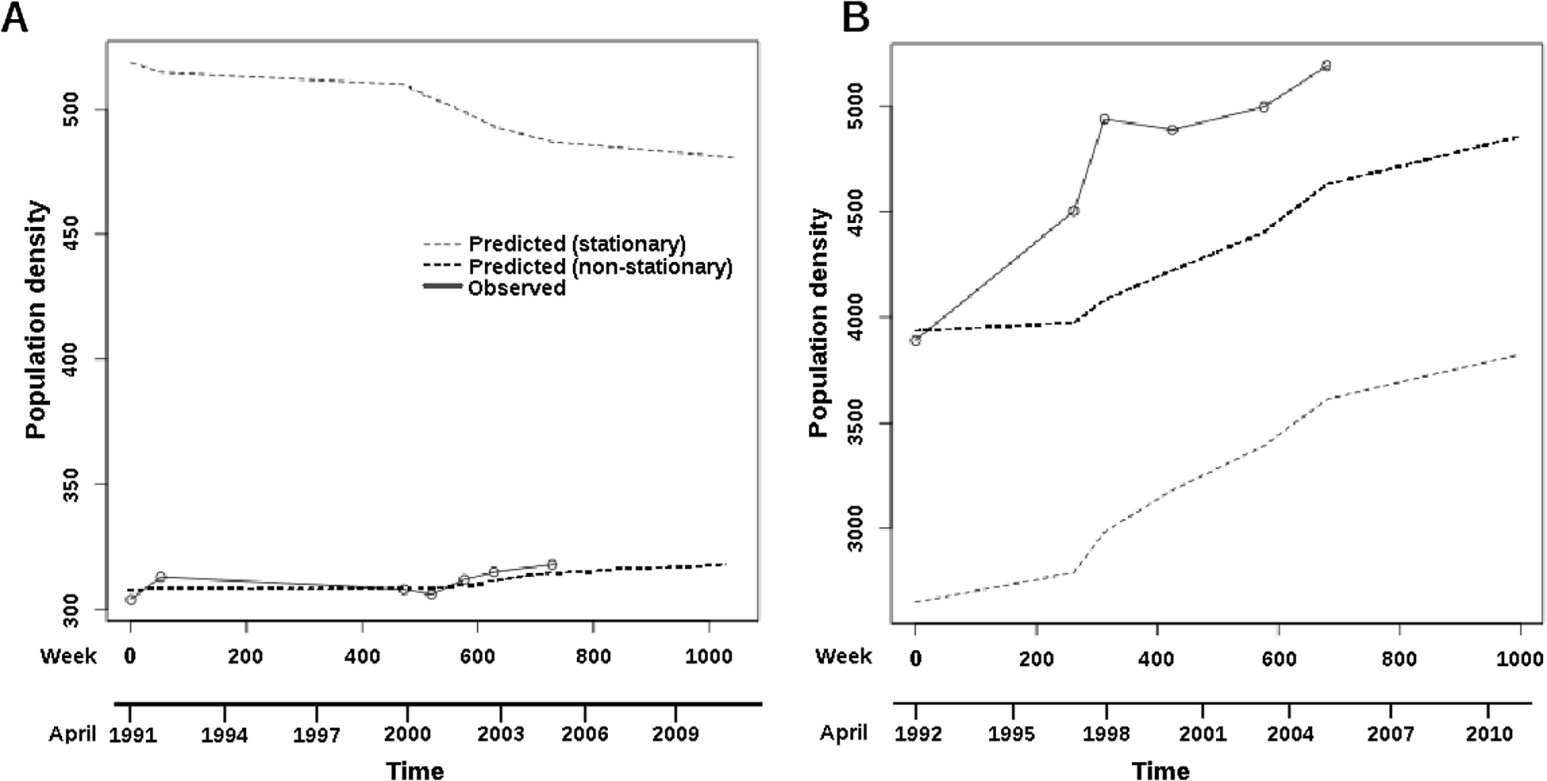
**Plots of population density against sampling time for A, Ban Khi Lek and B, Mahaxai.** The original observations are plotted, as well as GSS model predicted values for both the stationary and non-stationary cases. Time was measured in weeks, but an additional scale in years is given for convenience.

Independent runs of REML searches were found to converge on the same parameter estimates and REML values, regardless of the starting parameters (which were either the output of a run of ML searches (each based on the output of the previous search) or random variants around the LLR and SEG estimates). Consequently, it is likely that the parameter estimates reported (Table 3) are the best estimates or close to them. As with the SLR and GLM models, the population growth rate at MHX appears to be greater than that at BKL (Figure 2), and the error terms at BKL are both lower than those at MHX.

**Table 3:** REML parameter estimates for the “best-fit” GSS model found by SAN. Outlier values were excluded for the analyses

**Population**	**mu**	**ssq**	**tsq**	**exp(X0)**	**ln(REML)**
BKL	5.6178e-05	3.6003e-06	3.7835e-05	305.1172	32.4920
MHX	4.1704e-04	1.4548e-05	7.7833e-04	3925.056	22.8641

The positive likelihood values although unusual are not necessarily problematic. The SAN and Nelder-Mead functions used in the optimizations involve a normal probability density function (pdf) that can be very narrow and so go to very high values (i.e., > 1). It is conceivable that a tall, narrow, continuous pdf can cause the positive likelihoods here.

### Hypothesis testing

Frequency distribution plots were made for the simulated and bootstrap mu values to confirm symmetry, and thus the validity of our C.I.s, which assume a symmetrical distribution for mu. The C.I. for MHX (whether based on empirical data or on bootstrapped data) did not overlap the equivalence region, but in fact lay entirely above it (Table 4). Consequently, the hypothesis of non-equivalence could not be rejected (at α = 5 %) and a substantial positive growth trend appears to be present (at least between the years 1992 and 2005). In contrast, although the hypotheses of non-equivalence can also be rejected for BKL, the trend present could be either positive or negative because the equivalence region (i.e., that corresponding to a true mu value of zero) is entirely encompassed by the C.I.s for the estimated mu.

**Table 4:** Confidence intervals (95 %) for estimates of mu, with the “best-fit” GSS model, based on the original (empirical) data set, from bootstrapped data sets and from simulations (where mu = 0)

**Population**	**mu (empirical)**	**C.I. for mu (empirical)**	**C.I. for simulations**	**C.I. bootstrapped data**
BKL	5.6178e-05	−1.1812e-04 to 2.3048e-04	−1.2157e-07 to 1.1975e-07	−1.0721e-05 to 1.2234e-04
MHX	4.1704e-04	1.5234e-05 to 8.1885e-04	−3.9497e-06 to 3.9043e-06	1.3536e-04 to 5.3538e-04

Starting X values for the simulations were the observations made at t = 0 (i.e. in 1991 and 1992 for BKL and MHX, respectively).

### Predictions

Using the REML parameter estimates, and under the GSS model, the Kalman filter was employed to predict the 2011 population density for both the stationary and non-stationary cases (Table 5). This prediction is based on observed densities up to and including the 2005 observation, and assumes the same growth trend operates from 2006 to 2011 as operated between 1991/2 and 2005. Similar predictions were also made using the SLR and GLM (Poisson) models, where statistically appropriate. The predictions for all sample years, including 2011, are plotted in Figure 2 along with the original observations. At both BKL and MHX the population density changes appear best described by a non-stationary GSS model, particularly at BKL (e.g., much narrower C.I.s for the 2011 predictions in Table 5). The stationary GSS model for BKL indicates population decline rather than growth, but failed to model observed population changes between 1991 and 2005 very closely (Figure 2A). In all cases (including the SLR and GLM models) the observed densities at BKL and MHX in 2011 lay far below the lower tail of the 95 % C.I.s; this suggested that the population densities observed in 2011 are significantly less than those expected based on past population trends.

**Table 5:** Model-based predictions of the 2011 population densities

**Population**	**SLR**	**GLM (Poisson)**	**GSS (non-stationary)**	**SS (stationary)**
BKL	316.8257 ± 16.5514	316.8899 ± 42.3489	307.6708 ± 2.9599	480.8264 ± 31.3784
MHX	5854.6060 ± 851.7450	5995.6860 ± 268.7633	4394.885 ± 188.748	3822.493 ± 558.468

Values are given ± a 95 % confidence interval. The observed densities were 142 m^-2^ at BKL and 0 m^-2^ at MHX.

## Discussion

The results of the study indicate relatively stable population growth through the 1990s and early 2000s with a significant decline in population density between 2005 and 2011 at Mahaxai. Similarly at Thathot, near to the regulating pond of the NT2 dam, *N. aperta* population densities in 2011 were observed to have fallen to around 20 % of their 2004 levels. Unfortunately, no data for the period from 2006 to 2010 are available so that it is not possible to determine when the decline began, and in particular if it began only after the NT2 dam was closed in 2010. Nevertheless, together with other factors the impact of the NT2 impoundment should be evaluated as a potential cause of the decline in snail populations.

The eradication of *N. aperta* populations from the Xe Bang Fai river may have occurred before 2010; however, the river at, and downstream of, Mahaxai is no longer a suitable habitat for this snail (Additional File 1: figure S1). The mean flow rate in the river during the dry season (March to May) has been calculated to show an increase from 13 m^3^/s to around 140 m^3^/s after operation of the dam. In addition, the concentration of suspended solids during the low water period is expected to have increased from 45 to 95 mg/l. The diversion of Nam Theun waters into the Xe Bang Fai (via the downstream discharge channel) has led to increases in dry season river depth of 3.5 to 5 m above pre-impoundment levels [39]. The combination of increased current, turbidity and water depth would prevent *N. aperta* from becoming established in the Xe Bang Fai river downstream of the NT2 discharge channel. The impact of back-flow also appears to have eliminated habitats at least 5 km upstream, but areas further upstream have not been surveyed.

The decline in *N. aperta* population density at Ban Khi Lek, Northeast Thailand, 400 km downstream of the NT2 project may be part of a natural cycle or fluctuation because a drop in population density was seen in 2004, although this was not as marked as in 2011. The GSS model predicts a slight but steady decrease in population density from 1992 to 2001 with an upturn and population growth thereafter. In view of this, the population could be in a natural growth-decline cycle, but the density observed for 2011 is significantly less than that predicted by the model and much less than the observed 2001 value. The NT2 project could effect population reductions in the Mekong river, far downstream in Thailand in two main ways. First, the Xe Bang Fai river had much higher levels of calcium than the Nam Theun river (about one order of magnitude higher), with a total hardness of 131–149 mg/l (as CaCO3) recorded in February 1995 [40]. Consequently, the influx of Nam Theun waters, further diluted by rain water collected in the reservoir of the dam, will have lowered considerably the calcium content of the lower and middle Xe Bang Fai. Three main rivers, which drain central Laos, enter the Mekong upstream of Ban Khi Lek. The northern most is Nam Kading, then the Xe Bang Fai 150 km further South, and finally the Xe Banghieng about 2 km upstream of Ban Khi Lek and 200 km South of the confluence of the Mekong and Xe Bang Fai (Figure 1). Of these three rivers only the Xe Bang Fai drains a limestone platform and so this river is the main source of hard water flowing down the Mekong river through Ban Khi Lek. The snail *N. aperta* requires hard water to provide the calcium needed for rapid shell growth as populations re-establish in the Mekong river each year following the annual flood [32]. In view of this, it is possible that the dilution effect of the NT2 discharge into the Xe Bang Fai could be responsible for the decline in snail populations at Ban Khi Lek.

The NT2 project could also impact snail populations at Khemmarat through its effects on snail colonization. Past studies have indicated considerable gene-flow or genetic affinity between *N. aperta* populations of the lower Mekong river and the Xe Bang Fai river [10]. In addition, it has been proposed that *N. aperta* populations in the lower Mekong river are replenished following the annual flood by colonists from source populations in the lesser rivers of southern Laos. The hypothesis supported by the fact that the annual spate is less severe in these rivers and they tend to show greater snail population densities earlier in the dry season than habitats in the Mekong river itself [41]. The higher calcium levels, shallower waters and less severe spate may explain the much higher population densities at Mahaxai than at Ban Khi Lek. Consequently, the decline in population density at Ban Khi Lek could be due to a reduction in the upstream sources of snails from tributaries draining into the Mekong (i.e., the loss of the Xe Bang Fai populations).

Alternatively, the fall in population density could be attributed to operations of the sand dredging industry at 16°02′09′′; 105°16′55′′; this activity has led to increased silting at Ban Khi Lek and might lower snail population densities there. Sand dredging at Ban Khi Lek began around 2000 and it is unlikely that its activity would have a sudden and marked effect in 2011. Other factors that could explain the present observations are, an unreported snail control experiment at Ban Khi Lek in 2011, or some unknown polluting event such as a chemical spill from a factory in Laos or in Khemmarat. Snail population densities at Thathot also fell markedly between 2004 and 2011. Thathot is not directly affected by changes in flow caused by the NT2 project; this suggests that *N. aperta* populations in the region are declining for some reason unconnected with the NT2 project. Thathot is, however, close to the regulation pond of NT2 and the area may have been affected during construction by increased human activity in the area, such as increased numbers of people using the stream where the snails live for laundry, washing vehicles and other polluting activities. The NT2 project itself supports more people living in the area, through economic opportunities, and this alone could lead to increases in pollution of water bodies.

At both Mahaxai and Ban Khi Lek the GSS model appeared to fit the data best if non-stationarity was assumed. The finding implies that at both sites the snail populations were still growing towards the carrying capacity of their habitat. The explanation for this could be a recent *de novo* colonization of the river at Mahaxai and Ban Khi Lek or recovery from some past population crash; the latter is the most likely. It may be that *N. aperta* populations in these rivers are not stable in the long-term and experience regular local extinctions followed by recolonization events. If so, this would make the impacts of HEPPs more difficult to determine, in that they must be extracted from a background of natural population fluctuations.

## Conclusions

The present study did not find increases in *N. aperta* population densities downstream of the NT2 project as might be expected on the basis of experiences with schistosomiasis and HEPPs in other regions. In 2011 snail populations were observed to decline significantly, well below the levels predicted by trend analysis of the populations before the NT2 project went into operation. The NT2 HEPP could have caused this decline in snail abundance by increased river depth, current, silting and dilution of dissolved calcium. Falling snail population densities at Ban Khi Lek might also be linked to the loss of the Xe Bang Fai river populations, which may have been a source of colonists for recovery of the Thai Mekong river populations after the annual flood. Alternatively, the 2011 fall in snail density at Ban Khi Lek may have been due to sand dredging operations in the area or to pollution. The results also suggested that local population extinction and re-colonization may be a normal feature of *N. aperta* populations in these rivers, thus the fall in snail population density in the Mekong river of Thailand might be a result of natural population cycles present before the NT2 HEPP was initiated.

The findings show that the effects of HEPPs on *N. aperta* are certainly more complex than first thought and that much more data are required before reliable predictions can be made about the effects of these dams on Mekong schistosomiasis. The same data are also vital for the safe planning of new HEPPs in the region, so that the negative impacts of such water resource development on schistosomiasis control can be minimized.

Data which are most urgently required include, further monitoring of *N. aperta* populations in these and additional areas (such as upstream of Mahaxai) so that longer time series will become available, thus enabling the use of more conventional models that will improve predictions. Areas of the Nam Theun river (and associated water courses), which were not habitats for *N. aperta* prior to the NT2 HEPP, should also be surveyed because changes in water depth may have created new suitable habitats. In addition, more data are required on levels of turbidity and dissolved calcium in the areas affected in order to evaluate hypotheses regarding the impact of NT2 on population density. Finally, studies of the impact of other HEPPs (such as the Nam Theun-Hinboun project or the Pak-Mul Dam in Thailand) on *N. aperta* need to be improved and expanded to provide comparative data. The present report is based on a small set of observations, but it is the first step in providing much needed but currently lacking data of use in planning of HEPPs in the lower Mekong.

## Methods

### Sampling

Samples were taken at two sites in central Laos, from the Xe Bang Fai river at Mahaxai (17°24′45″N; 105°13′15″E) and from a spring flowing into the Nam Yom at Ban Thathot (17°37′30′′; 105°08′45′′), both in Khammouanne Province, and from one site in Northeast Thailand on the Mekong river at Ban Khi Lek (16°02′15′′; 105°18′00′′ ), Ubon Ratchathanee Province. All samples were taken within the low water period in the lower Mekong and within a five week period of each sampled year at Ban Khi Lek and an eight week period at Mahaxai (this was because the water level in the Xe Bang Fai river at Mahaxai is more stable over the low water period than in the Mekong at Ban Khi Lek). The duration of the survey was from 1991 to 2011 at Ban Khi Lek, from 1992 to 2011 at Mahaxai and from 2004 to 2011 at Thathot. A series of five flat rocks were collected from the river at each site (together comprising approximately 1 m^2^ in upper surface area) and placed under the water close to the shore at the same locations each year. The rocks were stored locally between sampling years so that only occasional replacement was required (due to loss or damage). The rocks were washed in river water and placed in the river for two days after which all *N. aperta* on the stones were washed into a tray and counted. The exact area for counting was marked out by scratching the rocks (to give 1 ± 0.00005 m^2^ sampling area) and snails outside the marked area were removed by brushing before the rocks were washed over the trays. Ecological studies have shown that periods in excess of two days did not result in any significant increase in snail density on rocks left in the river, and that stones preconditioned by exposure in the river for several days were no more or less attractive to the snails than washed and heat sterilized stones (in this study the latter were used) [42]. At Ban Khi Lek the rocks were placed in slightly different locations for samples in March and early April than for those taken later in the year; this was to ensure that the conditions of depth, current and insolation were the same for all samples. By placing the rocks in the same positions each year and following the same procedure, observation error between years was minimized. At Ban Thathot 8–12 smaller stones were collected, their surface areas measured, and the snail density per m^2^ estimated.

### Initial data analysis

All analyses in this study were performed using the R statistical package [43]. The data (snail counts per m^2^) were first screened for outliers. Any count that differed from the mean count (for all years sampled) by more than twice the corresponding standard deviation (SD) was classed as an outlier and was excluded from the final analyses. In order to check whether the data might be normally distributed a Shapiro-Wilk test was applied to both the original data and to the data with any outlier excluded. The sample dates were then converted to weeks, with the first sample being designated as week zero. In order to determine if the counts showed a simple linear relationship with time and to assess the effects of any outlier, a simple linear regression was performed for the data both with and without any outlier. Similarly, the fit of the data to a Poisson General Linear Model (GLM) was also assessed.

### The linear mixed model

Growth of the *N. aperta* populations sampled is modeled by fitting estimated population densities to a modified Gompertz State-Space exponential growth model (GSS) [38,44-46].

(1) $${\text{dX}}_{\text{t}}=\left(\text{ln}\lambda \right)\text{dt}+{\text{dB}}_{\text{t}}{\text{where dB}}_{\text{t}}∼\text{normal}\left(0,\sigma^{2}\right)$$

(2) $${\text{Y}}_{\text{t}}={\text{X}}_{\text{t}}+{\text{F}}_{\text{i}}{\text{where F}}_{\text{i}}∼\text{normal}\left(0,\tau^{2}\right)$$

The GSS can be seen to be an extension of the diffusion process model used in conservation biology [47].

The scaled GSS model has four unknown parameters, following the standard model of deterministic exponential growth:

(3) $$\beta \left(={\text{X}}_{0}\right),\mu \left(=\text{ln }\lambda \right),\sigma^{2}\text{and}\tau^{2}\text{,}$$

which are the hypothesized (unobserved) initial value of the (log) population density (at time t = 0), the expected change in density X_t_ over one (sampling) unit of time, a variance parameter representing process noise (environmental variability), and a variance parameter accounting for observation error. Y_t_ represents our observations at time t, whilst X_t_ represents the true population density that we cannot observe (only sample) [37]. The model assumes that the two sources of error or noise are uncorrelated and that neither is correlated with X. Values of X are likely to be correlated as, for example, a high population density in 2004 would increase the chance of a density as high or higher in 2005. The parameter μ or mu is the trend parameter, which is of most interest in the present analysis (specifically mu is the rate at which median population density changes over time).

Traditionally the GSS describes sigmoid growth, but here it is in the form of a linear, normal “state-space model”. The GSS model incorporates both observation (sampling) error and environmental process noise, and provides a likelihood function to link time-series population density estimates with the unknown parameters of the model [38]. In this sense it is superior to the two most commonly applied methods in time series analysis. These are the LLR model, log-linear regression of counts against time, and the Stochastic Model of Exponential Growth (SEG), which incorporates process noise and yields a log-normal probability distribution of population density. The LLR model implicitly assumes that observation error is the sole source of variation in the data (with population density governed by deterministic exponential growth), whilst the SEG assumes that variability in abundances is due entirely to growth rate fluctuations caused by environmental variability (process noise). Another popular approach to time series analysis is the Auto-Regressive, Integrated, Moving Average (ARIMA) approach; however, ARIMA was not considered suitable in this study because it requires sampling at 50 or more regular time intervals [48] although Y_t_ is a form of ARMA (Auto-Regressive, Moving Average) process in the present analysis. Recently methods have been developed to scale observations in the linear and mixed model framework to allow for unequally spaced time intervals [49] this approach has been applied to time series analysis with irregular sampling intervals, and found to perform well in comparison with the LLR and SEG models [37]. The scaled GSS approach has performed well with as few as five time samples [37]. Consequently, in view of the relatively short time series in the present study, irregular sampling intervals and that we could make few *a priori* assumptions about sources of error and noise, the scaled GSS model was chosen for the analysis of the present data.

### Parameter estimation

Initial values for the four parameters were taken from the LLR and SEG models after fitting the data; the initial value of μ (mu0) was the average of the estimate from the two models, the initial σ^2^ (ssq0) was from the SEG model, and initial τ^2^ (tsq0) and X_0_ (X00) from the LLR model (following published suggestions [37]). The fit of the scaled GSS model was maximized using the R script for the multivariate normal log-likelihood function provided in the literature [37], which uses Nelder-Mead optimization. Restricted maximum likelihoods (REMLs) are reported here in the final results because these have been shown to give improved estimation for the model [46]. REML values were calculated from the second differences of the successive population density samples following published methods [38]. Unlike ML, which optimized all four parameters, the REML procedure optimized only ssq and tsq (mu and X_0_ were then estimated from the error terms in light of the data). To avoid negative parameter values in iterations, σ^2^ and τ^2^ were log-transformed and the likelihoods maximized as if they were the true values.

It is known that GSS models can lead to a likelihood surface with multiple local maxima, such that the chance of detecting the true peak using a non-global method such as Nelder-Mead optimization is unlikely unless the initial parameter values are very close to this peak [50]. Two methods were used to overcome this problem. First, the model fitting was repeated 95000 times, with initial parameter values drawn from a normal distribution, with mean equal to the REML estimates from the first run (with starting values from the LLR and SEG models, as above) and SD set such that 95 % of the values drawn lay within 0.1 or 10 times the mean. The random number seed was also varied between runs. A REML frequency histogram was then plotted and the parameter estimates for models, corresponding to the minimum, mean, modal and maximum REML values, were inspected for relative variation and biological credibility. Second, a simulated-annealing approach linked to a Metropolis sampler (SAN) was used in an attempt to better explore the likelihood surface. Published studies had reported some success using the SAN approach in this context [38].

Simulated-annealing is a stochastic global optimization method [51]. The implementation in R was used, which applies the Metropolis function to obtain the acceptance probability. Default general settings were used, i.e., the proposal distribution was generated from a Gaussian Markov kernel with scale proportional to the actual temperature. Temperatures were decreased according to the logarithmic cooling schedule as given in Belisle (1992) [51]; specifically, the temperature was set to temp / log(((t-1) %/% tmax)*tmax + *e*), where t is the current iteration step and temp = 1 (10 for REML searches) and tmax = 200 (temp and tmax were chosen by running test chains of 5000000 iterations and examining the proposal acceptance rate and maximum likelihood, %/% denotes integer division). As implemented in this study, the algorithm will begin at a temperature of 1 or 10 and lower this value every 200 iterations, thus making it less probable that a bad move (i.e., one corresponding to parameter estimates with a lower likelihood) will be accepted as the chain progresses. Unlike Nelder-Mead, SAN is more global and is more likely to find the true maximum in cases where the likelihood surface is noisy, with many non-optimal peaks of similar magnitude to the true maximum [52]. An initial chain of 50000000 iterations was run with the LLR and SEG parameter estimates used as starting values, followed by three additional chains with starting values drawn from normal distributions, centered around the mean of the LLR/SAN estimates, as described above. In addition, different random number seeds were used for each chain. For each of these four runs, the resulting estimates were used as starting values for subsequent chains until the likelihood remained stable over at least 10000000 iterations. These searches used ML, rather than REML, because of its speed and greater proposal acceptance rate. The results of the ML searches were then used as starting values for REML searches. Four series of REML chains were also run *de novo*, with starting values obtained as with the initial ML runs.

### Making predictions and hypothesis testing

#### Testing for zero trend

The maximum REML model parameter values were used in subsequent hypothesis testing. First, a test for zero trend was performed using the standard error of the estimated mu and a standard normal percentile in an equivalence testing framework [53]. The null hypothesis being that a significant trend is present (i.e., the mu estimate lies outside a fixed, specified, interval containing zero). Traditionally this equivalence region is specified with reference to population data for the same or ecologically equivalent species; however, no such data were available for any similar population of freshwater snail. To substitute for an equivalence region, a null distribution was produced by simulating the data 5000 times, with mu fixed at zero and starting values of the remaining three parameters taken from the LLR and SEG models; the simulated data were then fitted to the scaled GSS model and the REML estimates of the parameter values recorded. The simulation procedure was that used by previous authors [37]. In this procedure the general stochastic version of the exponential growth model is used to simulate data with mu = 0, and on the log scale:

(4) $${\text{X}}_{\text{t}}={\text{X}}_{\text{t}-1}+\text{mu}+{\text{E}}_{\text{t}}{\text{where E}}_{\text{t}}∼\text{normal}\left(0\text{,}\;\sigma^{2}\right)$$

(5) $${\text{Y}}_{\text{t}}={\text{X}}_{\text{t}}+{\text{F}}_{\text{t}}{\text{ where F}}_{\text{t}}∼\text{normal}\left(0,\tau^{2}\right)$$

the simulated population densities are then exp(Y_t_).

A conventional 95 % confidence interval (±t. SE(mu)) was then estimated for the trend parameter. If the 95 % confidence interval for the estimated mu lay entirely outside that for the simulated mu values, then the null hypothesis that a substantial trend is present would be tentatively accepted. A 97.5 % confidence interval was obtained for the mu value estimated from the original data; this is equivalent to a 95 % confidence interval in the hypothesis tests, because the aim was not only to detect any significant difference, but also to consider the direction of that difference (i.e., greater or less than zero). This test procedure is not ideal because the same model is used to estimate mu as to simulate the data for the equivalence region; however, the method provides and objective estimate of the equivalence region and at least avoids the arbitrary decisions of conventional equivalence testing, as to what is an equivalent snail population and what the upper and lower bounds of the equivalence region should be.

In a similar manner a naïve bootstrap was used to estimate a sampling distribution for mu. The bootstrap was performed using 5000 replicates of the “sample()” function in R (i.e., simple sampling with replacement), the time series was then re-ordered by week, and if any two weeks (observations) had the same value, one week was added to the latter observation. The short length of the time series in the present study prevented the use of block bootstrapping to preserve time series structure and suggested little benefit in modeling the series using basis functions of continuous time (e.g., a Legendre polynomial). The bootstrapped sample should have the same distribution as the original sample; however, it must be noted that the empirical cumulative distribution function (cdf) may not be a good approximation to the true cdf as the time series here are short. Nevertheless, it is felt that the bootstrap approach provides a useful objective assessment of confidence for the present data. The bootstrap samples were used to estimate parameters with the scaled GSS model and a confidence interval for mu (estimated from the bootstrapped data) obtained as above.

#### Predicting the 2011 snail population density

A Kalman filter was used to yield optimized estimates of population density according to the scaled GSS model and maximum REML parameters obtained by the methods described above. The filter makes it possible to estimate the current value of the population process at time t (X_t_) under the scaled GSS model, given the history of the Y_t_ values up to and including Y_t _[38]. The R script used to implement the filter was taken from the literature [37]; however, this procedure assumes that the population under study is far from equilibrium (e.g., it is relatively recently established). The history of the *N. aperta* populations studied here is not known and the populations could have been fluctuating around the habitat's carrying capacity for some time. Consequently, the Kalman filter was modified to assume stationarity [38] and predictions for both stationary and non-stationary cases were made (here stationary refers to the joint distribution of all Y_t_). In the stationary case, the recursions of the filter were initiated with the mean and variance of the stationary distribution of Y_t_ i.e., a/(1-φ) and [σ^2^/(1-φ^2^) + τ^2^, where φ was obtained from the ARMA (AR1) equation for Y_t_.

For the case of non-stationary Y_t_, the recursions are initiated at m_o_ = X_o_ and variance = τ^2^, with X_o_ treated as an additional unknown parameter; this can describe a case in which the population itself is far from equilibrium and is still growing towards the carrying capacity [38].

Bootstrapping was used to generate a confidence interval for the predicted values, following the bootstrap procedure described above.

## Competing interests

The authors have no competing interests to declare.

## Authors’ contributions

ESU Jointly identified the need for this study and assisted with location, counting and identification of taxa and provided laboratory facilities and technical staff. SWA Jointly identified the need for this study, assisted with field work and performed the analyses. All authors read and approved the final version of the manuscript.

## Supplementary Material

Additional file 1 **Figure S1. Effect of discharge from the downstream channel of the Nam Theun 2 Dam on the environment of the Xe Bang Fai river at Mahaxai, Khammouanne Province, central Laos.** The two photos of the river at Mahaxai were both taken in late April and from approximately the same location. Photo A was taken in 2001 and photo B was taken in 2011. The Nam Theun 2 Dam began discharging water into the Xe Bang Fai just upstream of Mahaxai in October 2010. The yellow arrow heads indicate the same reference point in both photographs. Note the change in river depth and colour (indicating increased turbidity) and that the many islands present in 2001 were submerged in 2011. Click here for file
