# Supplementary figures and images for: Observations on Neotricula aperta (Gastropoda: Pomatiopsidae) population densities in Thailand and central Laos: implications for the spread of Mekong schistosomiasis
Source: Parasit Vectors. 2012 Jun 21;5:126. doi: 10.1186/1756-3305-5-126 (PMC3434010; doi:10.1186/1756-3305-5-126)

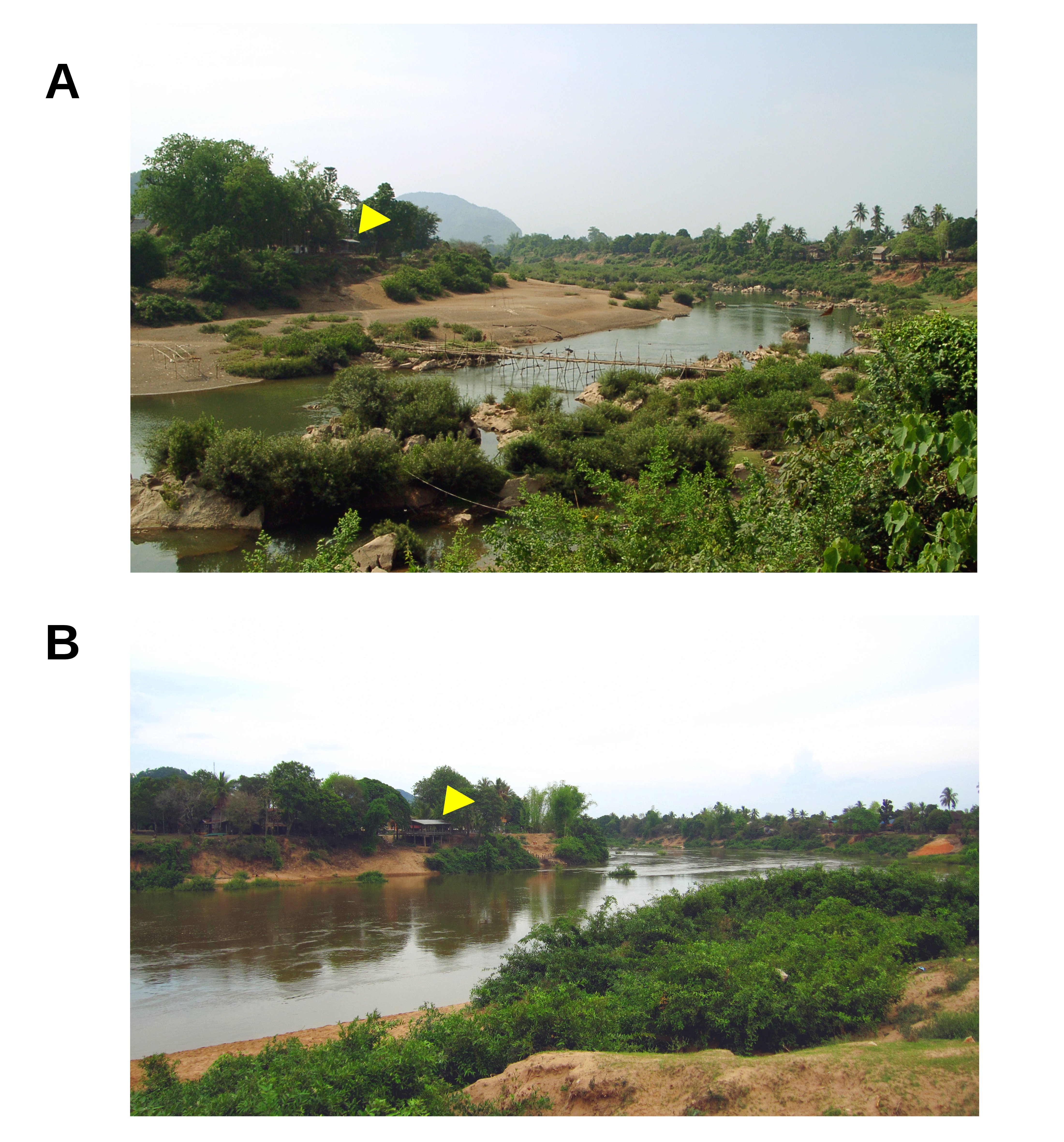

Supplement: Additional file 1 — Figure S1. Effect of discharge from the downstream channel of the Nam Theun 2 Dam on the environment of the Xe Bang Fai river at Mahaxai, Khammouanne Province, central Laos. The two photos of the river at Mahaxai were both taken in late April and from approximately the same location. Photo A was taken in 2001 and photo B was taken in 2011. The Nam Theun 2 Dam began discharging water into the Xe Bang Fai just upstream of Mahaxai in October 2010. The yellow arrow heads indicate the same reference point in both photographs. Note the change in river depth and colour (indicating increased turbidity) and that the many islands present in 2001 were submerged in 2011. [file 1756-3305-5-126-S1.jpeg]
